# Availability and Quality of Family Planning Services in the Democratic Republic of the Congo: High Potential for Improvement

**DOI:** 10.9745/GHSP-D-16-00205

**Published:** 2017-06-27

**Authors:** Dieudonné Mpunga, JP Lumbayi, Nelly Dikamba, Albert Mwembo, Mala Ali Mapatano, Gilbert Wembodinga

**Affiliations:** a Kinshasa School of Public Health, Faculty of Medicine, University of Kinshasa, Kinshasa, Democratic Republic of the Congo (DRC).; b Ministry of Public Health, Kinshasa, DRC.; c Lubumbashi School of Public Health, Faculty of Medicine, University of Lubumbashi, Lubumbashi, DRC.

## Abstract

**Objective:**

To determine the availability and quality of family planning services within health facilities throughout the Democratic Republic of the Congo (DRC).

**Methods:**

Data were collected for the cross-sectional study from April 2014 to June 2014 by the Ministry of Public Health. A total of 1,568 health facilities that reported data to the National Health Information System were selected by multistage random sampling in the 11 provinces of the DRC existing at that time. Data were collected through interviews, document review, and direct observation. Two dependent variables were measured: availability of family planning services (consisting of a room for services, staff assigned to family planning, and evidence of client use of family planning) and quality of family planning services (assessed as “high” if the facility had at least 1 trained staff member, family planning service delivery guidelines, at least 3 types of methods, and a sphygmomanometer, or “low” if the facility did not meet any of these 4 criteria). Pearson's chi-square test and odds ratios (ORs) were used to test for significant associations, using the alpha significance level of .05.

**Results:**

We successfully surveyed 1,555 facilities (99.2%) of those included in the sample. One in every 3 facilities (33%) offered family planning services as assessed by the index of availability, of which 20% met all 4 criteria for providing high-quality services. Availability was greatest at the highest level of the health system (hospitals) and decreased incrementally with each health system level, with disparities between provinces and urban and rural areas. Facilities in urban areas were more likely than in rural areas to meet the standard for high-quality services (*P*<.001). Public facilities were less likely than private facilities to have high-quality services (*P*=.02). Among all 1,555 facilities surveyed, 14% had at least 3 types of methods available at the time of the survey; the most widely available methods were male condoms, combined oral contraceptive pills, and progestin-only injectable contraceptives.

**Conclusion:**

Availability and quality of family planning services in health facilities in the DRC remain low, with inequitable distribution of services throughout the country. To improve access to and use of family planning, efforts should focus on improving availability and quality at lower health system levels and in rural areas where the majority of the population lives.

## Abstract (precis)

A few facilities provided good access to and quality of family planning services, particularly urban, private, and higher-level facilities. Yet only one-third offered family planning services at all, and only 20% of these facilities met a basic measure of quality. Condoms, oral contraceptives, and injectables were most available, whereas long-acting, permanent methods, and emergency contraception were least available. Responding to the DRC's high unmet need for family planning calls for substantial expansion of services.

## INTRODUCTION

Maternal and infant mortality remain high worldwide, especially in low-income countries.^1^^–^^3^ In the Democratic Republic of the Congo (DRC) specifically, in 2014 the estimated maternal mortality was 846 deaths per 100,000 live births and the estimated neonatal mortality was 28 per 1,000 live births.^4^ About 18% of women's deaths worldwide are due to preventable causes related to pregnancy and delivery, especially postpartum hemorrhage, hypertensive disorders, abortion, and sepsis.^1^^,^^5^^–^^10^ Three-quarters of these deaths could be prevented if health centers and hospitals provided a package of high-quality maternal care services.^11^^,^^12^ The risk factors of maternal and perinatal mortality are strongly entwined, with the first 24 hours of a newborn's life being those in which the risk of neonatal death is highest.^13^^,^^14^

Family planning is one of the most cost-effective interventions to improve maternal and child health outcomes^15^^–^^17^; it contributes to a reduction in the number of unwanted pregnancies, thus reducing the number of unsafe abortions and deaths from this cause. Family planning also reduces the *proportion* of pregnancies that are considered to be high risk—that is, pregnancies that occur too early or too late in relation to the mother's age, those that are spaced too closely together, or those that are considered high parity. By helping women time and space their pregnancies, family planning also helps ensure healthy nutritional outcomes for both mother and child.^18^ Raising women's awareness about family planning during antenatal care and childbirth and providing postpartum contraception during childhood vaccination visits are among the strategies that improve the use of family planning.^19^^,^^20^

Family planning is one of the most cost-effective interventions to improve maternal and child health outcomes.

The modern contraceptive prevalence rate in the DRC remains low at 8%, with significant disparities among provinces. The total fertility rate is high, estimated at 6.6 children per woman.^4^ Early childbearing among adolescents 15 to 19 years old also remains high, at 13 pregnancies per 1,000 girls. Approximately 27% of adolescent girls already have been pregnant or have given birth.^4^^,^^21^

The use of family planning is strongly related to both its availability and quality.^22^ In previous research conducted in Africa and Asia, the quality of family planning services tends to be higher in private than public facilities, and beneficiaries tend to be more satisfied with the quality of service provided by private health facilities compared with public providers.^22^^–^^24^ The quality of family planning is strongly influenced by the availability of trained human resources, materials, and equipment.^25^

According to the 2013–2014 Demographic and Health Survey (DHS) conducted in the DRC, unmet need for family planning was estimated at 27.7% among women in union and 43.0% among those not in union but sexually active. These high unmet need figures are due in part to limited access to quality family planning services in the country.^4^ The DRC faces several major challenges in meeting the family planning needs of its population, including the large land mass, which is the size of Western Europe, and poor supply chain management, which greatly hinders service delivery of any type of health service. The National Multisectoral Strategic Plan for Family Planning 2014–2020 set the objective of establishing family planning services in all 516 health zones of the country by 2020, but to date family planning services are available in less than half of those zones.^26^ In addition, provision of family planning services, including a range of contraceptive methods, are part of the minimum package of activities for all types of health facilities in the DRC.^27^^,^^28^

To date, no study has been conducted at the national level on the availability and quality of family planning services in the DRC—a major logistical feat given the physical expanse and poor transportation infrastructure of the country. The objective of this study was to determine the availability of family planning services within health facilities throughout the country and to assess their quality.

The objective of this study was to determine the availability and assess the quality of family planning services in health facilities throughout the DRC.

## METHODS

Data collection for this cross-sectional study was conducted from April 2014 to June 2014 by the Ministry of Public Health with technical and financial support from WHO and in collaboration with the Kinshasa School of Public Health.^29^

The health system in the DRC includes 4 types of facilities:

- Hospitals (including national and provincial hospitals, district hospitals, and secondary hospitals)
- Referral health centers
- Health centers
- Health posts

To be eligible for inclusion in this study, the facility had to be listed on the Ministry of Public Health roster of facilities and to have provided data to the National Health Information System (NHIS, known locally as SNIS) during the 6 months prior to the study as an indication that it was active.

Before selecting health facilities, the research team reviewed the list of facilities with health officials from each province and used the information to update the roster of facilities that were reporting to the NHIS. Only functional health facilities were included in the sampling frame. Each province was considered as a stratum, with 4 substrata corresponding to the 4 types of facilities. Because the proportion of health facilities providing family planning services in the DRC is not known, the sample size for the study was calculated considering a proportion (p) of 0.5 of health facilities having this characteristic of interest. The sample size was calculated for each of the 4 substrata and the selection of health facilities within the substratum was conducted by systematic random sampling using a sampling interval after arranging the health facilities in ascending order according to their national identification number. This procedure yielded a sample of 1,568 facilities. Additional details about the sampling procedures are provided in the full survey report.^29^

In view of the vast geographical expanse of the DRC and logistical challenges to collecting data, the research team divided the country into 38 “pools” corresponding to the major urban centers. The team contacted provincial health officials to determine the means of access to each selected facility and the resources needed to reach it.

### Dependent and Independent Variables

From the data collected, 2 dependent variables were created: an index of availability of family planning services and an index of quality of family planning services. These indices were calculated by modifying WHO-proposed tools to measure the preparation and availability of services.^30^

The **index of availability** was based on 3 criteria. A facility had to meet all 3 of these criteria to be considered as a facility that offered family planning services:

1. **Infrastructure:** existence of a room in which to provide family planning (and other) services that ensured the confidentiality and privacy of clients to be respected
2. **Staff:** existence of a health staff assigned to family planning services
3. **Service use:** evidence of client use of family planning services, based on service statistics (at least 1 client listed as obtaining family planning services in the 6 months preceding the survey)

The index of family planning availability was based on 3 criteria: infrastructure, staff, and service use.

The index of quality was informed by Donabedian's model of quality medical care.^31^ According to this model, there are 3 dimensions to judging quality: the structure of care in terms of inputs, material, staff, funds, and organizational structure; the processes used to deliver care (i.e., standards of care); and outcomes. The **index of quality** used in this study was based on 4 elements:

1. Presence of at least 1 staff member trained in family planning during the 2 years preceding the survey
2. Existence of family planning service delivery guidelines (printed manual of instructions or standards)
3. Availability of at least 3 types of contraceptive methods on the day of the survey (specifically, the 3 most widely used by clients, according to data from the 2013–14 DHS,^4^ which were male condoms, combined oral contraceptive pills, and injectable contraceptives)
4. Availability of a sphygmomanometer to measure blood pressure, which is desirable when prescribing certain contraceptive methods

The index of quality was based on 4 elements: presence of 1+ trained staff, existence of family planning service delivery guidelines, availability of 3+ methods, and availability of a sphygmomanometer.

These elements focused mostly on Donabedian's first dimension of quality care, which is focused on structure of care. Donabedian's second dimension, standards of care, was captured in our index by observing whether family planning service delivery guidelines existed. Since the elements included in our index comprised a modest measure of quality, only facilities that met all 4 of the criteria were classified as having “high” quality; if 1 or more of the criteria were not met, the facility was assessed as having “low” quality.

Independent variables included the health facility sector (public versus private), location of the facility (urban versus rural), type of facility (hospital, referral health center, health center, or health post), and province. Before carrying out statistical analyses, all non-state health facilities were grouped under the category “private,” which included private for-profit facilities, not-for-profit facilities, and church-managed facilities. Prior to 2015, the DRC had 11 provinces. In 2015, the provinces were further subdivided for a total of 26 provinces. This analysis is based on the 11 provinces in existence at the time of data collection.

### Data Collection and Analysis

Within each of the 38 pools, 2 staff from health facilities not selected for the study were recruited and trained by supervisors from Kinshasa as interviewers for the study. They visited all facilities selected for inclusion in that pool and collected data through structured interviews with managers and the person responsible for family planning services of health facilities. The first interviewer asked the questions and recorded the answers on a paper form while the second interviewer simultaneously recorded the information on an electronic form on a laptop computer. After the interview was completed, the 2 interviewers resolved any discrepancies between the paper and electronic forms. The interviewers also performed document review and directly observed conditions in the facilities (i.e., counting contraceptive products in stock, analyzing contraceptive use registers, availability of service delivery guidelines, and appearance of the consultation room). Supervisors revisited 10% of the facilities to validate the data.

Data were entered using CSPro 5.0, using double entry for quality control. All data were weighted by stratum before analysis. The data were then exported to Microsoft Excel 2010 to produce graphs and charts. SPSS Statistics version 21.0 and WINPEPI version 11.54 were used for analysis and testing of associations. The indices of availability of family planning and quality of services were calculated as a proportion of all facilities. Pearson's chi-square test or the Fisher exact test were used to test the association of different variables. The odds ratios (ORs) helped to measure the effect size of specific associations. All hypotheses were tested using the alpha significance level of .05.

### Ethical Review

The study was reviewed and approved by the Ethics (Human Subjects) National Committee. The research team obtained authorizations from national and provincial health authorities prior to the survey. Data were collected anonymously, after obtaining informed consent from participants.

## RESULTS

In total, 1,555 facilities of the 1,568 included in the sample (99.2%) were successfully surveyed across the 11 former provinces of the DRC. The 13 health facilities for which data were not collected were extremely difficult to access, with some requiring use of a motorized canoe.

### Availability of Family Planning Services Among the Total Sample of 1,555 Facilities

Of the 1,555 facilities surveyed, 33.0% offered family planning services, as assessed by the index of availability (i.e., availability of a room for family planning service provision, existence of staff assigned to family planning services, and evidence of client use of family planning services from service statistics) (Table 1). Hospitals were more likely to offer family planning (53.1%) than referral health centers (38.5%), health centers (31.1%), or health posts (8.9%). This relationship was statistically significant (*P*<.001). Availability of family planning was also significantly higher in urban areas than in rural areas (*P*=.02). By contrast, there was no relationship between the availability of family planning and whether the facility was in the public or private sector (*P*=.37).

**TABLE 1:** Percentage of Health Facilities With Family Planning Services, as Defined by the Availability Index,^a^ DRC, 2014 (N=1,555 Facilities)

	No. of Health Facilities	Family Planning Services Available^a^ No. (%)	*P* Value
Total	1,555	513 (33.0)	
Type			<.001
Hospitals	433	230 (53.1)	
Referral health centers	244	94 (38.5)	
Health centers	498	155 (31.1)	
Health posts	380	34 (8.9)	
Sector			.37
Public	872	296 (33.9)	
Private	683	217 (31.8)	
Location			.02
Urban	367	140 (38.1)	
Rural	1,188	373 (31.4)	

Abbreviation: DRC, Democratic Republic of the Congo.

a Index of availability: (1) availability of a room for family planning service provision; (2) existence of staff assigned to family planning services; and (3) evidence of client use of family planning services from service statistics.

Only 33% of surveyed facilities offered family planning services, as assessed by the index of availability.

**FIGURE 1:**
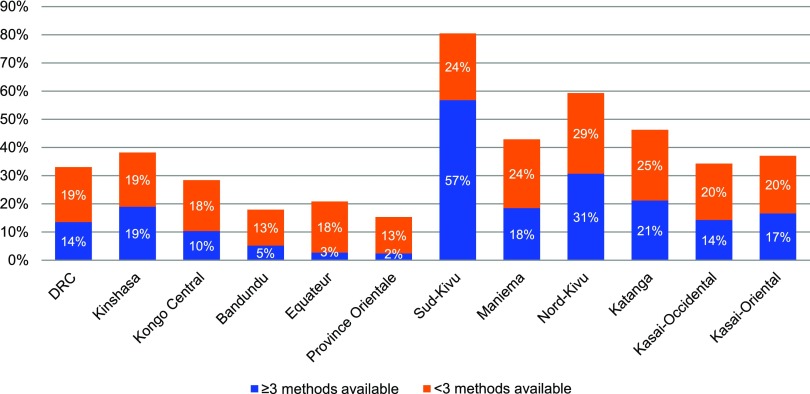
Percentage of Health Facilities With Family Planning Services Available by Province, Broken Down by Those With 3+ Methods Available, DRC, 2014 (N=1,555 Health Facilities Nationally) Abbreviation: DRC, Democratic Republic of the Congo.

As shown in Figure 1, family planning service availability was unevenly distributed across the country. The provinces with the highest percentages were Sud-Kivu (81%) and Nord-Kivu (60%), in contrast to the lowest percentages in province Orientale (15%), Bandundu (18%), Equateur (21%), and Kongo Central (28%). The bars in Figure 1 also indicate the percentage of facilities at the national level and in each province with 3 or more methods available (indicating greater choice) versus fewer than 3 methods. At the national level, of the 33% of facilities with family planning services, more had fewer than 3 methods (19%) while a few number of facilities had 3+ methods (14%). Sud Kivu and Nord Kivu had the highest percentage of facilities with 3+ methods, in stark contrast to Bandundu, Equateur, and Province Orientale (with 5% of health facilities or less having 3+ methods).

Family planning service availability was unevenly distributed across the country.

**FIGURE 2:**
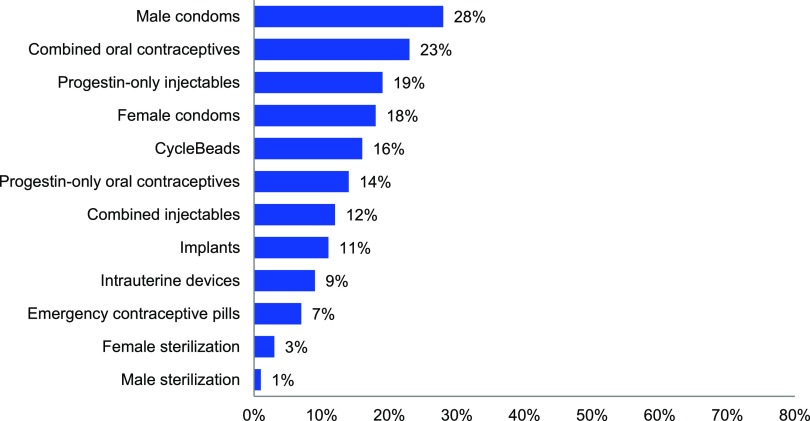
Percentage of Health Facilities With Specific Contraceptive Methods in Stock at the Time of the Survey, DRC, 2014 (N=1,555 Facilities^a^) Abbreviation: DRC, Democratic Republic of the Congo. ^a^ Data are based on the full sample of 1,555 health facilities surveyed, not the subset that had family planning services available as defined by the index of availability.

The relative availability of different contraceptive methods is evident from Figure 2. Based on the total of 1,555 facilities surveyed, the 3 most commonly available methods were condoms (28%), combined oral contraceptives (23%), and injectables (19%). Methods available in less than 10% of facilities were the intrauterine device (IUD), emergency contraception, and female and male sterilization. The availability of specific methods was higher in private than public facilities for implants, IUDs, emergency contraception, and female sterilization, but not significantly different for other methods (data not shown).

The 3 most commonly available methods available were condoms, combined oral contraceptives, and injectables.

### Quality of Family Planning Services Among the 513 Facilities With Family Planning Available

**FIGURE 3:**
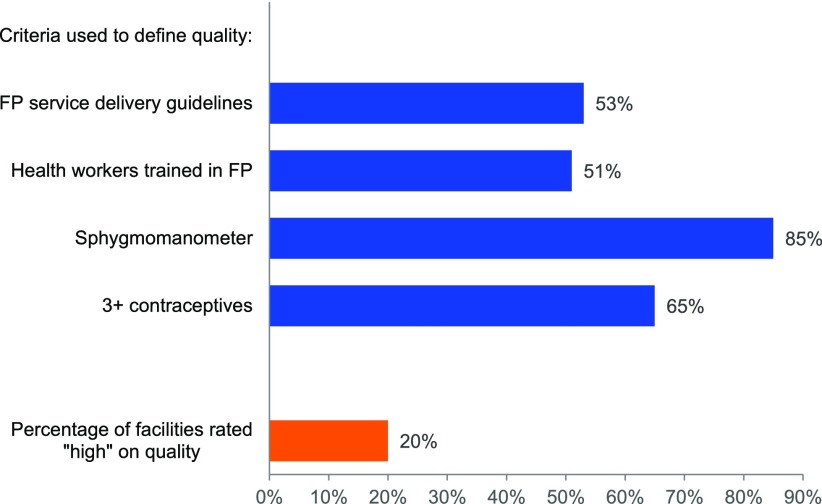
Percentage of Health Facilities With Family Planning Services Available That Met the Criteria for High Quality, by Element and in Total, DRC, 2014 (N=513 Health Facilities) Abbreviation: DRC, Democratic Republic of the Congo.

In this analysis, we developed a quality index based on 4 items for the 513 health facilities with family planning services available. As shown in Figure 3, just over half of these health facilities had service delivery guidelines (53%) and staff trained in family planning (51%). The large majority (85%) had a sphygmomanometer in good condition, and about 65% had at minimum male condoms, combined oral contraceptive pills, and injectable contraceptives available. However, only 1 of every 5 health facilities (20%) met all 4 quality criteria and thus met the standard of having a “high” quality of family planning services.

Only 20% of facilities were assessed as having high-quality family planning services.

**FIGURE 4:**
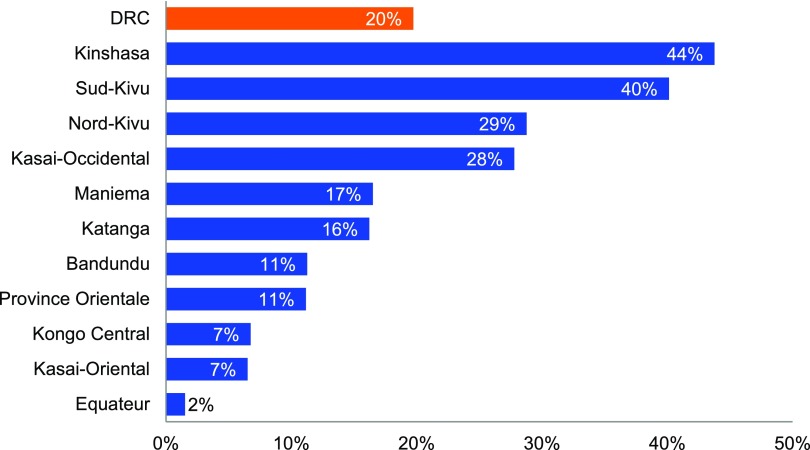
Percentage of Health Facilities With Family Planning Services Available That Were Rated “High” on Service Quality,^a^ Nationally and by Province, DRC, 2014 (N=513 Health Facilities) Abbreviation: DRC, Democratic Republic of the Congo. ^a^ Facilities were assessed as providing high-quality family planning services if they met all 4 criteria of the quality index: (1) availability of at least 1 staff trained in family planning in the prior 2 years; (2) existence of service delivery guidelines in family planning; (3) availability of, at minimum, male condoms, combined oral contraceptive pills, and injectables; and (4) availability of a sphygmomanometer.

The quality findings show marked differences by province (Figure 4). The facilities with the highest percentages offering high-quality family planning services were in Kinshasa (44%), Sud-Kivu (40%), Nord-Kivu (29%) and Kasai-Occidental (28%). By contrast, in the remaining provinces, 17% of facilities or less were judged to be of high quality.

According to the results in Table 2, the percentage of health facilities assessed to have high quality of family planning services was significantly higher for urban (35.1%) than rural areas (14.8%). It was also significantly higher among private health facilities (25.0%) than public (16.4%). Finally, quality was highest among hospitals (27.5%) and lowest among health centers (14.8%) and health posts (0.0%).

**TABLE 2.** Quality of Family Planning Services by Health Facility Characteristic, DRC, 2014 (N=513 Health Facilities)

	High Quality No. (%)	Low Quality No. (%)	*P* Value
Total	103 (20.1)	410 (79.9)	
Type			<.001
Hospitals	63 (27.5)	166 (72.5)	
Referral health centers	17 (18.1)	77 (81.9)	
Health centers	23 (14.8)	132 (85.2)	
Health posts	0 (0.0)	35 (100.0)	
Sector			.02
Public	48 (16.4)	245 (83.6)	
Private	55 (25.0)	165 (75.0)	
Location			<.001
Urban	47 (35.1)	87 (64.9)	
Rural	56 (14.8)	323 (85.2)	

Abbreviation: DRC, Democratic Republic of the Congo.

The number of methods available is important in family planning services because it serves as a proxy for the choice that clients have (more being better). The 513 facilities with family planning services available had a mean number of 3.0 methods available (Table 3). The number was higher for referral health centers (4.9) and national or provincial hospitals (4.7) than for health centers or posts (1.6).

**TABLE 3:** Number of Contraceptive Methods Offered by Type of Health Facility, DRC, 2014 (N=513 Facilities With Family Planning Service Availability)

No. of Contraceptive Methods	Type of Health Facility (%)			
	National and Provincial Hospitals^a^(n=10)	District Hospitals and Referral Health Centers^a^ (n=217)	Health Centers and Posts (n=286)	All Health Facilities(N=513)
0	30.0	34.8	67.9	53.4
1–2	20.0	4.6	6.3	5.7
3–4	0.0	5.4	8.8	7.3
≥5	50.0	55.2	17.1	33.6
Mean number	4.7	4.9	1.6	3.0

Abbreviation: DRC, Democratic Republic of the Congo.

a In the DRC, hospitals are classified into 3 categories: national, provincial, and district.

The 513 facilities with family planning services available had a mean number of 3 methods available.

## DISCUSSION

This study demonstrates the acute lack of access to quality family planning services in the DRC. Only 1 in 3 health facilities had family planning services available (defined as having a room in which family planning services could be provided, staff assigned to family planning services, and evidence of client use of family planning services). And of these facilities, only 1 in 5 were assessed to have “high” quality services (defined as having family planning service delivery guidelines, health workers trained in family planning in the past 2 years, a sphygmomanometer, and at least 3 types of contraceptive methods). These results confirm those found in 2012 through a stakeholders' mapping in maternal, newborn, and child health that also found family planning services were available in only approximately one-third of health facilities.^32^

This study demonstrates the acute lack of access to quality family planning services in the DRC.

Unmet need for family planning in the DRC is high among both women in union and sexually active women not in union.^4^^,^^33^ The low availability and quality of family planning services found in this study highlights the need for the country to improve access to and availability of services to better meet the demand for family planning.

In addition, we found that family planning services are more available in hospitals than in health centers and in urban than rural areas in the DRC. These differences could explain the persistence of low modern contraceptive prevalence of 8% in the country, with significant disparities between urban (15%) and rural areas (5%) and between provinces.^4^^,^^32^ In the DRC, the majority of the population lives in rural areas where health care is mainly provided through health centers. In this study, the majority of the provinces which had a higher percentage of facilities with family planning service (Sud-Kivu, Nord-Kivu, Katanga, Maniema, Kinshasa, and Kasai-Oriental) are among those that have a large number of urban cities.

Family planning services are more available in hospitals than in health centers and in urban than rural areas in the DRC.

The National Multisectorial Strategic Plan for Family Planning 2014–2020 in the DRC defines the quality of family planning service as “high” when a health facility has at least 1 staff trained in family planning and provides a varied range of contraceptive methods, specifically at least 3 types of contraceptive methods comprising 1 long-acting method, 1 short-acting method, and 1 natural method.^26^ When looking specifically at the number of methods available, we found that 14% of all health facilities surveyed in our study had at least 3 types of contraceptive methods; although the 3 minimum methods defined in our study were all short-acting methods (condoms, pills, and injectables), this definition is consistent with the standards proposed by the National Program of Reproductive Health.^34^ Although this figure is low, it seems that progress has nevertheless been made over the past 2 years, with the percentage of health facilities offering at least 3 types of contraceptive methods increasing from 6% in the 2012 mapping study^32^ to 14% in our study.

Male condoms, combined oral contraceptive pills, and progestin-only injectable contraceptives were the most available methods in health facilities in our study; these results are similar to those found in the 2012 stakeholders' mapping study as well as by Utoo and colleagues in Nigeria.^32^^,^^35^ We think the predominance of condoms highlighted in these studies may be due to their use in other programs such as HIV and sexually transmitted infection control programs. In contrast, other recent studies have found high popularity of long-acting reversible contraceptives (LARCs) in several countries of Africa and Asia. For example, Jesse Rattan et al., reporting on an initiative to increase the availability, quality, and use of contraceptives in crisis-affected settings in Chad and the DRC, found a dramatic and sustained increase in use of implants and IUDs; these methods became among the most used methods in both country settings.^36^ Similarly, Munroe et al., using data from social franchising programs in 17 African and Asian countries (not including the DRC), showed the majority of the clients chose LARCs.^37^ Rattan and colleagues reported on an intervention project focused mainly in eastern DRC during the last humanitarian crisis, which likely explains the difference in high availability of LARCs in that study and low availability in our study. A greater percentage of private than public health facilities in our study offered LARCs. Private facilities thus provide a significant portion of family planning services in the DRC, especially as LARCs may be increasingly preferred by sexually active women of childbearing age compared with short-acting methods. To reach the goal of 19% of modern contraceptive prevalence outlined in the National Multisectoral Strategic Plan for Family Planning 2014–2020,^26^ the Government should strengthen partnerships with private providers, despite the fact that private health facilities are inequitably distributed between urban and rural areas.^27^

We also showed that facilities in urban areas are more likely than those in rural areas to meet the quality standard, as are private facilities compared with public facilities. The quality of family planning deteriorated when moving down the health system chain, from hospitals to health posts. These results are consistent with those found in the multicenter study by Hutchinson et al. in Ghana, Kenya, and Tanzania, which showed that the quality of the family planning offered by private providers was better than that offered by public providers,^23^ particularly when comparing primary-level health facilities. Private health facility users liked the short waiting times and infrequent shortages of inputs.^22^^,^^23^ These results corroborate the finding that health services that are under the direct responsibility of public administrations often raise problems of poor resource management.

Facilities in urban areas are more likely than those in rural areas to meet the quality standard, as are private facilities compared with public facilities.

Kayembe et al. reported in 2015 that the capability of health facilities in Kinshasa, the capital city of the DRC, to provide quality family planning services had improved between 2012 and 2013, with clinics offering higher-quality family planning services than hospitals and health centers.^38^ The number of years in operation and the number of available methods were linked to these improvements. In our study, there were overall few clinics integrated into the NHIS, resulting in a small sample size. Based on our numbers, we found that only 44% of health facilities in Kinshasa met the quality standard, compared with 68% of clinics in the Kayembe report. The difference is likely due to differences in the way quality was measured between the 2 studies and in different sampling approaches.

Low availability and quality of family planning are among the main reasons for the low contraceptive prevalence in the DRC, but not the only reasons. Demand for family planning among the population also needs to be taken into account. For example, the majority of the population in the DRC is influenced by religious leaders who often are opposed to family planning. Moreover, in developing countries overall and in the DRC specifically, many people hold “pronatalist” views and are therefore hesitant to use family planning. To improve access to and use of family planning, health officials need to address both supply and demand considerations.

### Strengths and Limitations

This study is the first to assess progress toward increasing access to and quality of family planning services on a national scale in the DRC. Also, it was methodologically stronger than the previous 2012 stakeholder mapping because of the sampling technique used, which involved stratification by province and type of health facility leading to greater representativeness of all types of health facilities. In addition, one of the strengths of this study is that it used a systematic sampling strategy and collected data representing the whole country despite important challenges in this context.

Data collection for this study covered approximately 10% of health facilities integrated into the NHIS of the DRC. The main limitation is that the sampling excluded health facilities not integrated into the NHIS, which could have led to selection bias, leading to an overestimation of family planning service availability and quality. However, we believe facilities not reporting to the NHIS receive less support and thus the availability and quality of family planning services is likely to be poor. On the other hand, we did not consider certain practices, such as the use of hangars for family planning service provision (an outside location with only a roof overhead), in our index of availability of family planning services because confidentiality and privacy of the clients would be difficult, if not impossible, under these circumstances. In addition, pharmacies—the major source of contraception in the 2007 and 2013–14 DHS studies—were excluded from this study. These facts may have resulted in an underestimation of family planning service availability. Finally, the measurement of quality was based on the availability of pills, condoms, and injectables, because they were the most widely reported methods; in contrast, other studies of this type base the measure of “at least 3 methods” on the availability of *any* type of modern method.

## CONCLUSION

Availability and quality of family planning services in the DRC remain low. Family planning services are inequitably distributed throughout the country, with better availability in urban than rural areas and with significant differences in availability between provinces. Although efforts have been made to improve the availability of family planning services in selected rural areas, given the vast number of rural health zones that are still lacking family planning interventions, services are more available in urban areas than in rural areas where the majority of the population lives. Private health facilities are likely to provide better quality family planning services than public health facilities. Health authorities should work toward strengthening public-private partnerships to achieve improved access to and quality of care in family planning services.

## Notes

**Peer Reviewed**
